# Investigating the impact of type 2 diabetes mellitus on brain function in obstructive sleep apnea patients using regional homogeneity and seed-based functional connectivity methods

**DOI:** 10.3389/fnins.2025.1581884

**Published:** 2025-08-20

**Authors:** Wang Zhezi, Xiong Yanxi, Xie Xingwu, Li Sheng, Mo Bencheng, Su Guangping, Lv Yunyi, Liu Yue

**Affiliations:** Department of Radiology Imaging Center, Renmin Hospital, Hubei University of Medicine, Shiyan, Hubei, China

**Keywords:** T2DM, OSA, cognitive impairment, regional homogeneity, functional connectivity, resting-state functional magnetic resonance imaging

## Abstract

**Objective:**

This study aims to investigate the effects of Type 2 Diabetes Mellitus (T2DM) on brain function in patients with Obstructive Sleep Apnea (OSA) using Regional Homogeneity (ReHo) combined with seed-based Functional Connectivity (FC) methods.

**Materials and methods:**

46 OSA patients, 38 OSA with T2DM patients, and 34 healthy controls (HC) were prospectively recruited. Clinical data were collected from all participants, and neuropsychological testing was performed using the Montreal Cognitive Assessment (MoCA), Mini-Mental State Examination (MMSE), and Epworth Sleepiness Scale (ESS). Resting-state functional magnetic resonance imaging (rs-fMRI) data were collected, and ReHo combined with seed-based FC analysis was used to assess brain function differences among the three groups. Finally, partial correlation analysis was conducted to investigate the relationship between clinical variables and imaging metrics ofthe differential brain regions.

**Results:**

Compared to HCs group, the OSA group showed increased ReHo in the left occipital gyrus, and decreased ReHo in the right fusiform gyrus and left cerebellum region 8. Furthermore, FC between the left occipital gyrus and left cerebellum region 8, as well as between the right fusiform gyrus and left cerebellum region 3, was significantly decreased. Partial correlation analysis revealed a significant negative correlation between ReHo in the right fusiform gyrus and the Oxygen Desaturation Index (ODI), and a significant positive correlation between FC in the left cerebellum region 8 and MMSE scores. Compared to the OSA group, the OSA with T2DM group exhibited decreased ReHo in the left occipital gyrus, with increased FC between the left occipital gyrus and left thalamus. Partial correlation analysis showed that ReHo in the left occipital gyrus was significantly negatively correlated with the Insulin Resistance Index (IRI), while FC in the left thalamus was negatively correlated with MoCA scores and positively correlated with hemoglobinA1c (HbA1c) levels.

**Conclusion:**

T2DM affects brain function in OSA patients, further exacerbating cognitive burden. These findings provide valuable insights into the neuropathological mechanisms ofT2DM in OSA and support the development of objective neuroimaging biomarkers.

## 1 Introduction

Type 2 diabetes mellitus (T2DM) is a metabolic disorder characterized by hyperglycemia and hyperinsulinemia. With improvements in living standards and an aging population, the global number of individuals with T2DM is projected to reach 642 million by 2024 (Yang et al., 2024). Prolonged hyperglycemia and glycemic variability in T2DM contribute to systemic macrovascular and microvascular complications, which can ultimately lead to severe multi-organ comorbidities (Xu et al., 2024). As a chronic metabolic condition, T2DM is associated with reduced expression of insulin receptors and decreased receptor tyrosine kinase activity, which promotes the accumulation of amyloid-*β* (Aβ) and tau proteins (Arnold et al., 2018). Chronic hyperglycemia and insulin resistance impair neuronal function, synaptic plasticity, and neurovascular integrity, triggering neuroinflammation and oxidative stress that exacerbate brain injury and eventually result in cognitive impairment (Van Sloten et al., 2020). Obstructive sleep apnoea (OSA) is a highly prevalent sleep-related breathing disorder. Based on the criterion of an apnea-hypopnoea index (AHI) ≥ 5 episodes per hour, approximately 936 million individuals aged 30–69 years worldwide are affected by OSA (Benjafield et al., 2019). OSA is characterized by recurrent partial (hypopnea) or complete (apnea) upper airway obstruction during sleep, accompanied by sleep disturbances (such as fragmentation or deprivation), intermittent hypoxia, hypercapnia, and other inflammation-related comorbidities. Furthermore, in terms of cognitive function, individuals with OSA exhibit varying degrees of impairment compared to healthy controls (Mohammadi et al., 2025).

Multiple cross-sectional studies suggest a potential bidirectional relationship between OSA and T2DM. On one hand, OSA may exacerbate glucose metabolic dysfunction through various mechanisms. Firstly, two hallmark features of OSA—intermittent hypoxia and sleep fragmentation—can activate the sympathetic nervous system, thereby promoting insulin resistance (IR) and increasing hepatic glucose production (Pamidi and Tasali, 2012). Secondly, intermittent hypoxia has been shown to be a risk factor for glucose metabolism disorders, independent of obesity. Recurrent cycles of hypoxia and reoxygenation can trigger oxidative stress and promote the release of inflammatory cytokines such as TNF-*α* and IL-6. The resulting systemic inflammatory state further contributes to the dysregulation of adipokines such as leptin and adiponectin, ultimately impairing insulin signaling pathways (Drager et al., 2011; Ryan et al., 2005). Thirdly, chronic sleep deprivation and poor sleep quality significantly impair glucose homeostasis, as evidenced by reduced insulin sensitivity and compromised compensatory capacity of pancreatic *β*-cells. Moreover, sleep deprivation can activate the hypothalamic–pituitary–adrenal axis, leading to increased cortisol secretion and gluconeogenesis, thereby exacerbating fasting hyperglycemia (Spiegel et al., 2009). On the other hand, the metabolic abnormalities associated with T2DM may impair respiratory control and upper airway stability, increasing the risk of OSA. Studies have reported that the prevalence of OSA among individuals with T2DM ranges from 24 to 86% (West et al., 2018). Compared with individuals without T2DM, those with T2DM are nearly twice as likely to develop OSA, and this association is independent of potential confounding variables and conventional OSA risk factors (Subramanian et al., 2019). A prospective cohort study further demonstrated that T2DM patients with elevated fasting insulin levels and higher homeostasis model assessment of insulin resistance (HOMA-IR) scores have a significantly increased incidence of OSA (Huang et al., 2022). Although there is a strong association between OSA and T2DM, most current neuroimaging studies continue to examine them as separate conditions. Given their high comorbidity and the potential synergistic effects on neurocognitive function, it is essential to investigate how the coexistence of OSA and T2DM specifically impacts brain function.

In this study, we included a healthy control group, an OSA group, and an OSA with T2DM group, and applied regional homogeneity (ReHo) and functional connectivity (FC) analyses to examine the impact of T2DM on brain function in OSA. ReHo assesses the temporal synchronization between a given voxel and its neighboring voxels, servin as an important indicator of local neuronal activity (Yu et al., 2023). FC measures the temporal correlation of neural activity between spatially separated brain regions, allowing for the assessment of functional interactions between impaired regions and other brain areas (Han et al., 2025). We hypothesize that T2DM may aggravate brain dysfunction in OSA patients and contribute to increased cognitive burden.

## 2 Materials and methods

### 2.1 Participants

This study employed a prospective recruitment design. From June 2023 to November 2024, we screened the medical record system of Shiyan People’s Hospital, affiliated with Hubei University of Medicine, and enrolled 46 OSA patients, 38 OSA with T2DM patients, as well as 34 age-, sex-, and education-matched healthy controls (HCs). The inclusion criteria for the OSA group were as follows: (1) age between 30 and 70 years; (2) right-handedness; (3) at least 6 years of formal education; and (4) a diagnosis of moderate-to-severe OSA, with an AHI > 15 events/h according to the 2.3 edition (2017) of the American Academy of Sleep Medicine (AASM) Manual for the Scoring of Sleep and Associated Events (Kapur et al., 2017), with no history of continuous positive airway pressure (CPAP) treatment. For the OSA with T2DM group, the inclusion criteria were: (1) age between 30 and 70 years; (2) right-handedness; (3) at least 6 years of formal education; (4) a confirmed diagnosis of T2DM base on the 2010 diagnostic criteria of the American Diabetes Association (ADA) (American Diabetes A, 2010), with stable long-term treatment; (5) a diagnosis of moderate-to-severe OSA (AHI > 15 events/h) according to Version 2.3 of the scoring manual by the American Academy of Sleep Medicine (AASM, 2017), and medical records indicated that the diagnosis of OSA preceded the onset of T2DM by at least 12 months. Clear information on the duration of T2DM (in years) was provided to be used as a covariate in subsequent analyses. The exclusion criteria applied to all three groups were as follows: (1) presence of other sleep disorders; (2) diabetic complications such as retinopathy, nephropathy, or peripheral neuropathy; (3) type 1 diabetes mellitus, history of diabetic ketoacidosis, hyperosmolar state, or severe hypoglycemia; (4) cerebrovascular disease, traumatic brain injury, or structural brain abnormalities detected by routine MRI; (5) history of cardiovascular or psychiatric disorders; (6) thyroid dysfunction, severe hepatic or renal impairment, infectious diseases, or other somatic conditions that may affect cognition; (7) history of alcohol abuse, substance abuse, or use of psychotropic medications; (8) contraindications to MRI; (9) left-handedness or ambidexterity.

This study was conducted in accordance with the Declaration of Helsinki and was approved by the Ethics Committee of Shiyan People’s Hospital. Written informed consent was obtained from all participants.

### 2.2 General information

General demographic and clinical information, including sex, age, body mass index (BMI), years of education, and duration of diabetes, was collected using a standardized patient questionnaire.

### 2.3 Clinical indicators and neuropsychological assessment

Clinical indicators collected from all three groups included systolic and diastolic blood pressure, triglycerides, total cholesterol, high-density lipoprotein, low-density lipoprotein, AHI, oxygen desaturation index (ODI), minimum oxygen saturation (SaO₂), total sleep time (TST), sleep efficiency, fasting blood glucose, insulin resistance index (IRI), and hemoglobinA1c (HbA1c). All participants underwent neuropsychological assessments, including the Montreal Cognitive Assessment (MoCA), the Mini-Mental State Examination (MMSE), and the Epworth Sleepiness Scale (ESS). These assessments were conducted by trained resident physicians with more than 2 years of clinical experience, under the supervision of a senior physician, and completed within 3 h prior to MRI scanning.

### 2.4 Polysomnography

All patients underwent polysomnography within 3 days prior to MRI scanning using the Compumedics Grael system (Australia). Participants were instructed to abstain from consuming any beverages known to affect sleep, including tea, coffee, and alcohol, during the 24 h preceding the examination. Each monitoring session lasted for at least 8 h, with the start time tailored to each participant’s habitual sleep schedule. All polysomnography data were interpreted by board-certified attending physicians with more than 5 years of experience and specialized training.

### 2.5 MRI acquisition

Structural and functional MRI data were acquired from all participants using a 3.0 T superconducting MRI scanner (MAGNETOM Skyra, Siemens Healthcare, Erlangen, Germany) equipped with a 32-channel phased-array head coil. Participants were instructed to lie supine, remain awake, minimize focused mental activity, and keep their heads still with the help of foam padding. All MRI scans were performed by professionally trained radiology residents with over 2 years of experience. High-resolution structural images were acquired using a sagittal three-dimensional magnetization-prepared rapid gradient echo (3D-MPRAGE) sequence with the following parameters: repetition time (TR) = 2000 ms, echo time (TE) = 2.49 ms, flip angle = 7, slice thickness = 1.0 mm, field of view (FOV) = 256 × 256 mm, voxel size = 1 mm × 1 mm × 1 mm, total slices = 192, and scan time = 4 min 40 s. Rs-fMRI was acquired using a blood oxygenation level-dependent gradient recalled echo-echo planar imaging (BOLD GRE-EPI) axial sequence with the following parameters: TR = 2000 ms, TE = 30 ms, flip angle = 90, number of slices = 33, slice thickness = 4.0 mm, FOV = 240 mm × 240 mm, voxel size = 3 mm × 3 mm × 4 mm. A total of 240 volumes were obtained over a scan duration of 8 min 8 s.

### 2.6 Rs-fMRI data preprocessing

Resting-state fMRI data were preprocessed using RESTplus_V11.2.^1^ The preprocessing steps included: (1) conversion of DICOM files to NIfTI format; (2)excluding the first 10 time points; (3) slice timing correction; (4) head motion correction (participants with translation >2 mm or rotation >2 ° were excluded); (5) spatial normalization with resampling to 3 mm × 3 mm × 3 mm voxel size; (6) linear trend removal; (7) nuisance covariate regression (including white matter, gray matter, cerebrospinal fluid signals, and Friston-24 motion parameters); (8) bandpass filtering.

### 2.7 ReHo calculation

After preprocessing, ReHo maps were calculated based on the Kendall’s coefficient of concordance (KCC), which assesses the similarity of the time series among each voxel and its 26 neighboring voxels. A standardized ReHo map was obtained by dividing the local KCC by the mean KCC of the whole brain. To enhance the signal-to-noise ratio, the standardized ReHo maps were smoothed with a 6 mm × 6 mm × 6 mm Gaussian kernel. Finally, the ReHo values of brain regions showing significant group differences were extracted for each participant using RESTplus for further correlation analyses.

### 2.8 FC analysis

Based on the results from section 1.7, brain regions showing significant ReHo differences were selected as seed regions. A voxel-wise whole-brain FC analysis was then performed by calculating the Pearson correlation coefficients between the mean time series of the seed region and the time series of every other voxel in the brain. The resulting correlation coefficients were transformed into Z-scores using Fisher’s r-to-Z transformation. Finally, two-sample *t*-tests were conducted at the voxel level to compare FC patterns across groups.

### 2.9 Statistical analysis

All statistical analyses were performed using SPSS version 26.0. For continuous variables, data conforming to a normal distribution are expressed as mean ± standard deviation, while non-normally distributed data are presented as median and interquartile range. Categorical data are expressed as percentages. Group comparisons of demographic and clinical variables were conducted using one-way analysis of variance (ANOVA) or the Kruskal–Wallis test, with a significance threshold set at *p* < 0.05. *Post-hoc* analysis were conducted when significant group effects were found to identify specific group differences. To examine group differences in whole-brain ReHo and FC metrics, ANOVA was conducted using sex, age, and head motion parameters as covariates. Group differences in ReHo were considered statistically significant if they survived Gaussian Random Field (GRF) correction with a voxel-level threshold of *p* < 0.005 and a cluster-level threshold of *p* < 0.05. For FC, statistical significance was defined as voxel-level *p* < 0.01 and cluster-level *p* < 0.05 after GRF correction. Finally, partial correlation analyses were conducted to examine the associations between imaging metrics and clinical characteristics, PSG parameters, as well as neuropsychological test scores, with sex, age, BMI, years of education, and diabetes duration (in years) included as covariates. A *p*-value of < 0.05 was considered statistically significant.

## 3 Result

### 3.1 Demographic characteristics

Three participants were excluded due to excessive head motion (translation >2 mm or rotation >2°), resulting in a final sample of 1 15 participants: 46 in the OSA group, 35 in the OSA with T2DM group, and 34 in the HCs group. Table 1 presents the demographic characteristics of the three groups and the results of *post-hoc* analysis. A significant group difference was observed in BMI (*p* = 0.000), whereas no significant differences were found in sex, age, or years of education (all *p* > 0.05).

**Table 1:** Comparison of demographic characteristics.

Characteristics	OSA with T2DM group (*n* = 35)	OSA group (*n* = 46)	HCs group (*n* = 34)	Statistical value	*p* value
Gender (M/F)	9/26	18/28	14/20	2.20	0.333
Age (years)	51.20 ± 8.65	48.52 ± 12.81	49.82 ± 14.35	0.48	0.621
BMI(kg/m^2^)	28.55 ± 3.30	26.40 ± 2.94	25.53 ± 3.08	8.85	0.000^b^
Education (years)	11.00(5.00, 16.00)	10.00(4.00, 16.00)	10.00(9.00, 12.00)	0.77	0.465
Diabetes duration(years)	5.00(1.00,30.00)	—	—	—	—

OSA, obstructive sleep apnoea; T2DM, 2 diabetes mellitus; HCs, healthy controls; BMI, body mass index. ^b^Significant difference between HCs group and OSA with T2DM group in post-hoc analysis.

### 3.2 Comparison of clinical characteristics, PSG scores, and neuropsychological assessments

Table 2 summarizes the clinical characteristics and post hoc analysis among the OSA, OSA with T2DM, and HCs groups. Significant group differences were observed in triglycerides (TG), total cholesterol (TC), low-density lipoprotein (LDL), apnea–hypopnea index (AHI), oxygen desaturation index (ODI), nadir SaO₂, mean SaO₂, percentage of time with SaO₂ < 90%, N1 and N3 sleep stages, MMSE, MoCA, and ESS. No significant differences were found among the three groups in diastolic blood pressure (DBP), systolic blood pressure (SBP), high-density lipoprotein (HDL), TST, sleep efficiency, N2 stage, or rapid ice moment (REM) sleep.

**Table 2:** Assessment of clinical features, PSG scores, and neuropsychological test scores.

Characteristics	OSA with T2DM group (*n* = 35)	OSA group (*n* = 46)	Hc group (*n* = 34)	Statistical value	*p* value
DBP (mmHg)	119.09 ± 8.43	119.48 ± 10.28	116.35 ± 10.66	1. 12	0.33
SBP (mmHg) TG (mmol/L)	74.60 ± 8.83	74.35 ± 9.46	71.85 ± 6.74	1.2	0.306
TC (mmol/L) HDL (mmol/L) LDL (mmol/L)	2.82 ± 2.07	2.44 ± 1.71	1.80 ± 0.75	3.45	0.035b
AHI/ (events/h) ODI/ (events/h) Nadir SaO2 (%)	4.30 ± 3.60	3. 11 ± 2.02	2.86 ± 1. 13	3.34	0.032b
Mean SaO2 (%)	1.67 ± 0.81	1.66 ± 0.61	1.57 ± 0.75	0. 19	0.826
SaO2 < 90%	3.58(1.04, 19.30)	2.72(1.84, 15.30)	2.09(0.84,2.95)	5.63	0.005bc
TST(min)	36. 17 ± 17.76	19.09 ± 3.98	2.39 ± 0.74	96.23	0.000abc
Sleep efficiency (%)	27.71 ± 23.52	17.68 ± 10.09	1.21 ± 0.64	29.58	0.000abc
N1 (%)	80.00(50.00,90.00)	85.00(50.00,93.00)	96.00(90.00,99.00)	50. 17	0.000ab
N2 (%)	92.00(87.00,98.00)	90.00(86.00,98.00)	95.50(90.00,98.00)	17.84	0.000ab
N3 (%)	13.20(3.50,37.20)	16.20(3.60,36.30)	1.40(0.30,3.50)	39.48	0.000ab
REM (%)	504.56 ± 71.87	512.79 ± 83.58	506.5 ± 77.82	0. 12	0.884
MMSE	85.30(58.30,95.30)	87.70(56.60,98.30)	87.35(56.60,98.30)	0.22	0.802
MoCA	30.40(15.00,45.40)	30. 10(10.30,44.90)	9.85(6.70, 14.30)	93.02	0.000ab 0.912
ESS	28.50(18.50,51.20)	29.50(17.40,48.50)	29.40(18.50,47.50)	0. 10	0.000abc 0.058
FBG (mmol/L) HOMA-IR	26.60(4.60,37. 10)	15.60(5.70,36.70)	35.60(29.30,39.40)	32.79	0.000abc
HbA1c (%)	11.80(4.20,27.40)	13.60(4.80,39.00)	18.95(5.70,32.50)	2.9	0.000abc
	21.48 ± 3.95	24.41 ± 2.71	28.91 ± 1.38	58.91	0.000ab
	21.06 ± 3.46	23.48 ± 3.30	28.74 ± 1.42	62.04	—
	17.00(14.00,20.00)	17.00(14.00,20.00)	2.00(1.00,6.00)	713.4	—
	8.40(5.30,22.62)	—	—	—	—
	5. 18(2.24,8.74)	—	—	—	
	11. 10(4. 10, 13.20)	—	—	—	

OSA, obstructive sleep apnoea; T2DM, 2 diabetes mellitus; HCs, healthy controls; DBP, diastolic blood pressure; SBP, systolic blood pressure; TG, triglyceride; TC, total cholesterol; HDL, high-density lipoprotein; LDL,low-density lipoprotein; AHI, apnea-hypopnea index; ODI, oxygen desaturation index; SaO2, oxygen saturation; TST, total sleep time; MMSE, mini-mental state examination; MoCA, montreal cognitive assessment; ESS, Epworth Sleepiness Scale; FBG, fasting blood glucose; HOMA-IR, homeostatic model assessment of insulin resistance; HbA1c, hemoglobinA1c. a, Significant difference between HCs group and OSA group in *post-hoc analysis*. b, Significant difference between HCs group and OSA with T2DM group in *post-hoc* analysis. c, Significant difference between OSA group and OSA with T2DM group in *post-hoc* analysis.

### 3.3 Group differences in ReHo and seed-based functional connectivity

**Figure 1:**
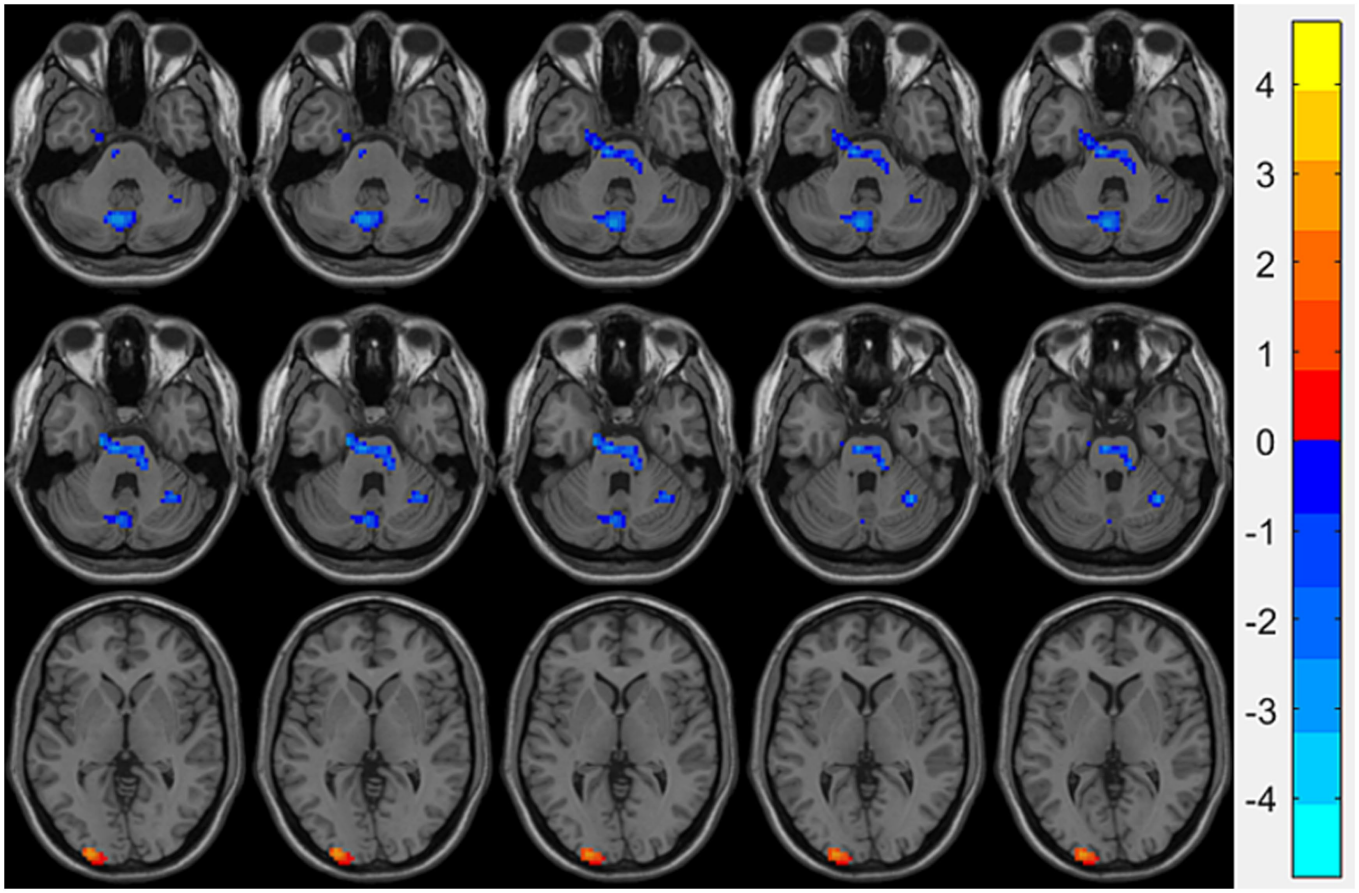
Brain regions showing significant ReHo differences between the OSA group and HCs. Cool-colored areas represent regions with significantly decreased ReHo values overlaid on axial slices of the MNI152 standard brain template, while warm-colored areas indicate regions with significantly increased ReHo values. The color bar represents *t*-values, with deeper colors indicating greater *t*-values. ReHo, Regional Homogeneity; MNI, Montreal Neurological Institute.

**Figure 2:**
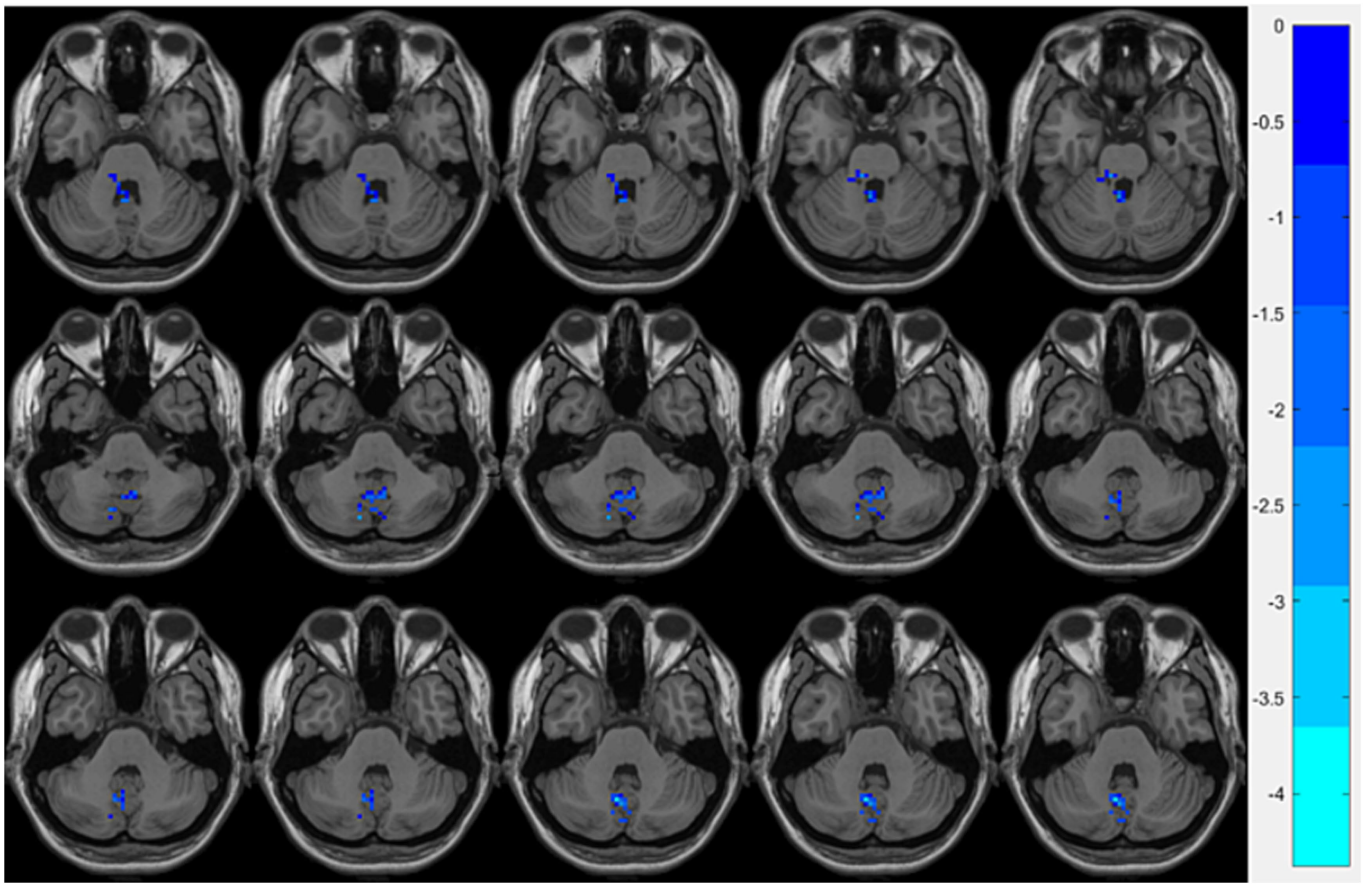
Brain regions showing significant FC differences between the OSA group and HCs. Cool-colored areas represent regions with significantly decreased FC values overlaid on axial slices of the MNI152 standard brain template. The color bar represents *t*-values, with deeper colors indicating greater *t*-values. FC, Functional Connectivity; MNI, Montreal Neurological Institute.

Compared with the HCs group, the OSA group showed significantly reduced ReHo values in the right fusiform gyrusand the left cerebellum region 8, while significantly increased ReHo values were observed in the left middle occipital gyrus (Table 3; Figure 1). Seed-based FC analysis using these regions revealed significantly decreased FC between the left middle occipital gyrus and the left cerebellum region 8, and between the right fusiform gyrus and the left cerebellum region 3 (Table 3; Figure 2).

**Table 3:** Significant differences in ReHo and seed-based FC between OSA group and HCs group.

Brain regions	Peak MNI coordinates			Peak T value	Voxels
	X	Y	Z		
ReHo					
OSA group<HCs group					
Fusiform_R	27	−9	−36	−4.5412	232
Cerebelum_ 8_L	−6	−69	−33	−4. 1,636	432
OSA group>HCs group					
Occipital_Mid_L	−27	−96	−3	4.368	224
FC					
OSA group<Hc group					
Cerebelum_ 8_L	−6	−63	−33	−4.2363	83
Cerebelum_3_L	−9	−33	−24	−4.3366	40

The brain region labels are derived from the Automated Anatomical Labeling (AAL116) atlas. ReHo represents regional homogeneity, FC represents functional connectivity. MNI refers to the Montreal Neurological Institute. The T value indicates statistical significance (*p* < 0.05, GRF correction). Fusiform_R, the right fusiform gyrus; Cerebelum_8_L, the left cerebellum region 8; Cerebelum_3_L, the left cerebellum region 3; Occipital_Mid_L, the left occipital gyrus; R, right; L, left.

**Figure 3:**
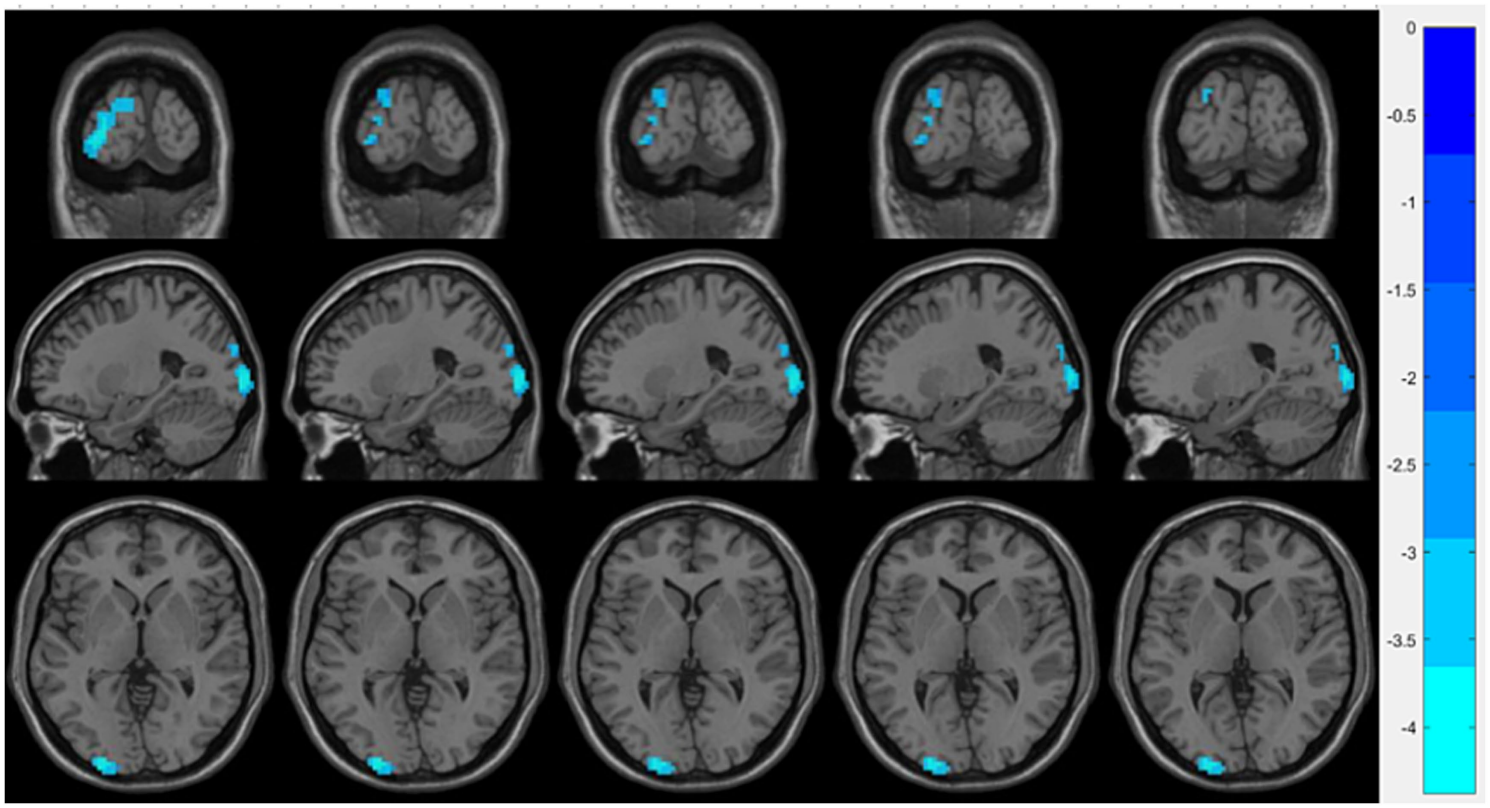
Brain regions showing significant ReHo differences between the OSA with T2DM group and the OSA group. Cool-colored areas represent regions with significantly decreased ReHo values overlaid on axial slices of the MNI152 standard brain template. The color bar represents *t*-values, with deeper colors indicating greater *t*-values. ReHo, Regional Homogeneity; MNI, Montreal Neurological Institute.

**Figure 4:**
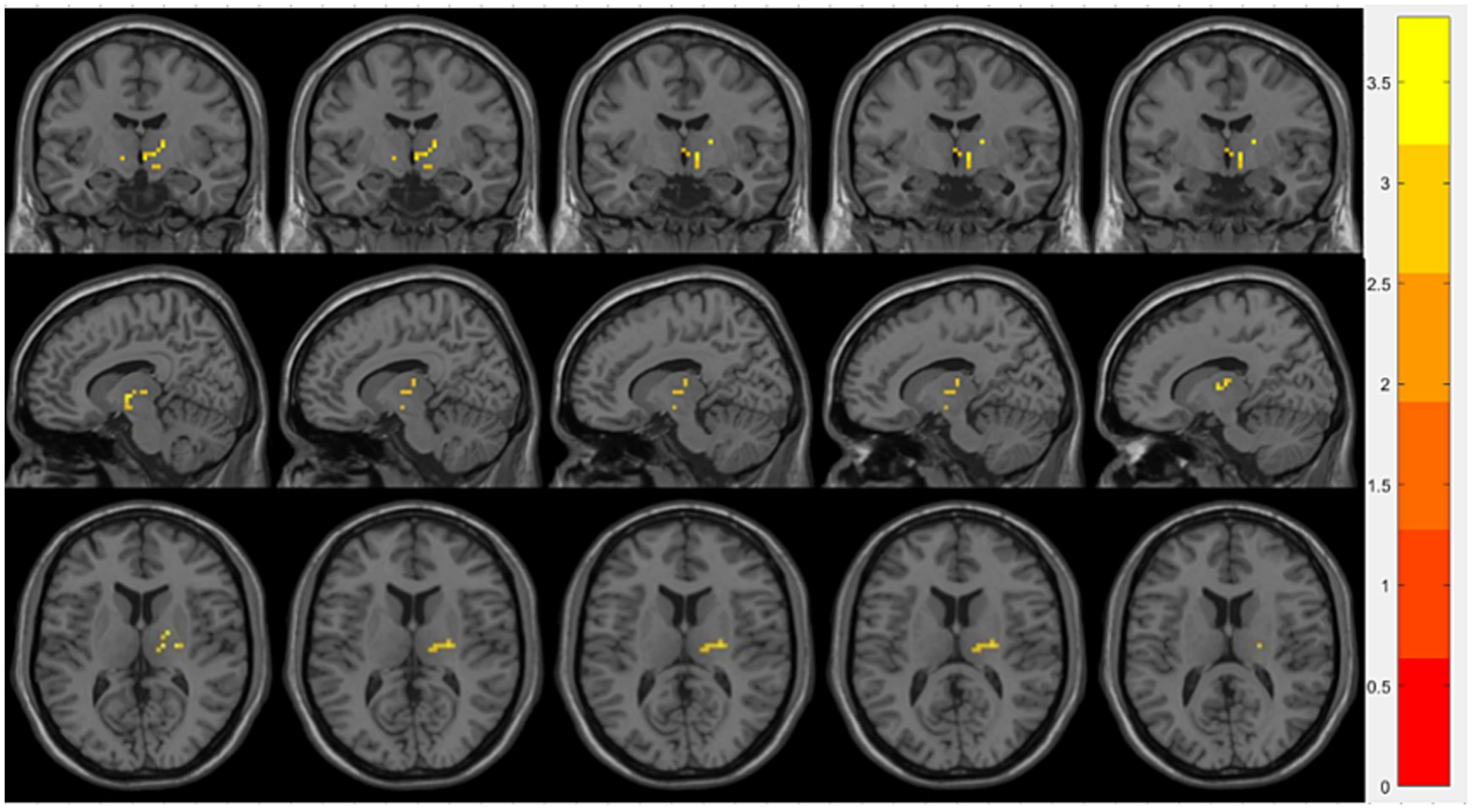
Brain regions showing significant FC differences between the OSA with T2DM group and the OSA group. Warm-colored areas represent regions with significantly increased FC values overlaid on axial slices of the MNI152 standard brain template. The color bar represents *t*-values, with deeper colors indicating greater *t*-values. FC, Functional Connectivity; MNI, Montreal Neurological Institute.

Compared with the OSA group, the OSA with T2DM group exhibited significantly reduced ReHo in the left middle occipital gyrus (Table 4; Figure 3). FC analysis using this region as a seed showed significantly increased FC between the left middle occipital gyrus and the left thalamus (Table 4; Figure 4).

**Table 4:** Significant differences in ReHo and seed-based FC between OSA with T2DM group and OSA group.

Brain regions	Peak MNI coordinates			Peak T value	Voxels
	X	Y	Z		
ReHo					
OSA with T2 DM group< OSA group					
Occipital_Mid_L	−27	−99	−3	−4.3821	171
FC					
OSA with T2 DM group> OSA group					
Thal_VPL_L	−15	−18	3	3.8262	54

The brain region labels are derived from the Automated Anatomical Labeling (AAL116) atlas. ReHo represents regional homogeneity, FC represents functional connectivity. MNI refers to the Montreal Neurological Institute. The T value indicates statistical significance (*p* < 0.05, GRFcorrection). Occipital_Mid_L, the left occipital gyrus; Thal_VPL_L, the thalamus ventral posterolateral nucleus; L, left.

The brain region labels are derived from the Automated Anatomical Labeling (AAL116) atlas. ReHo represents regional homogeneity, FC represents functional connectivity. MNI refers to the Montreal Neurological Institute. The T value indicates statistical significance (*p* < 0.05, GRF correction). Fusiform_R, the right fusiform gyrus; Cerebelum_8_L, the left cerebellum region 8; Cerebelum_3_L, the left cerebellum region 3; Occipital_Mid_L, the left occipital gyrus; R, right; L, left.

### 3.4 Partial correlation analysis

**Figure 5:**
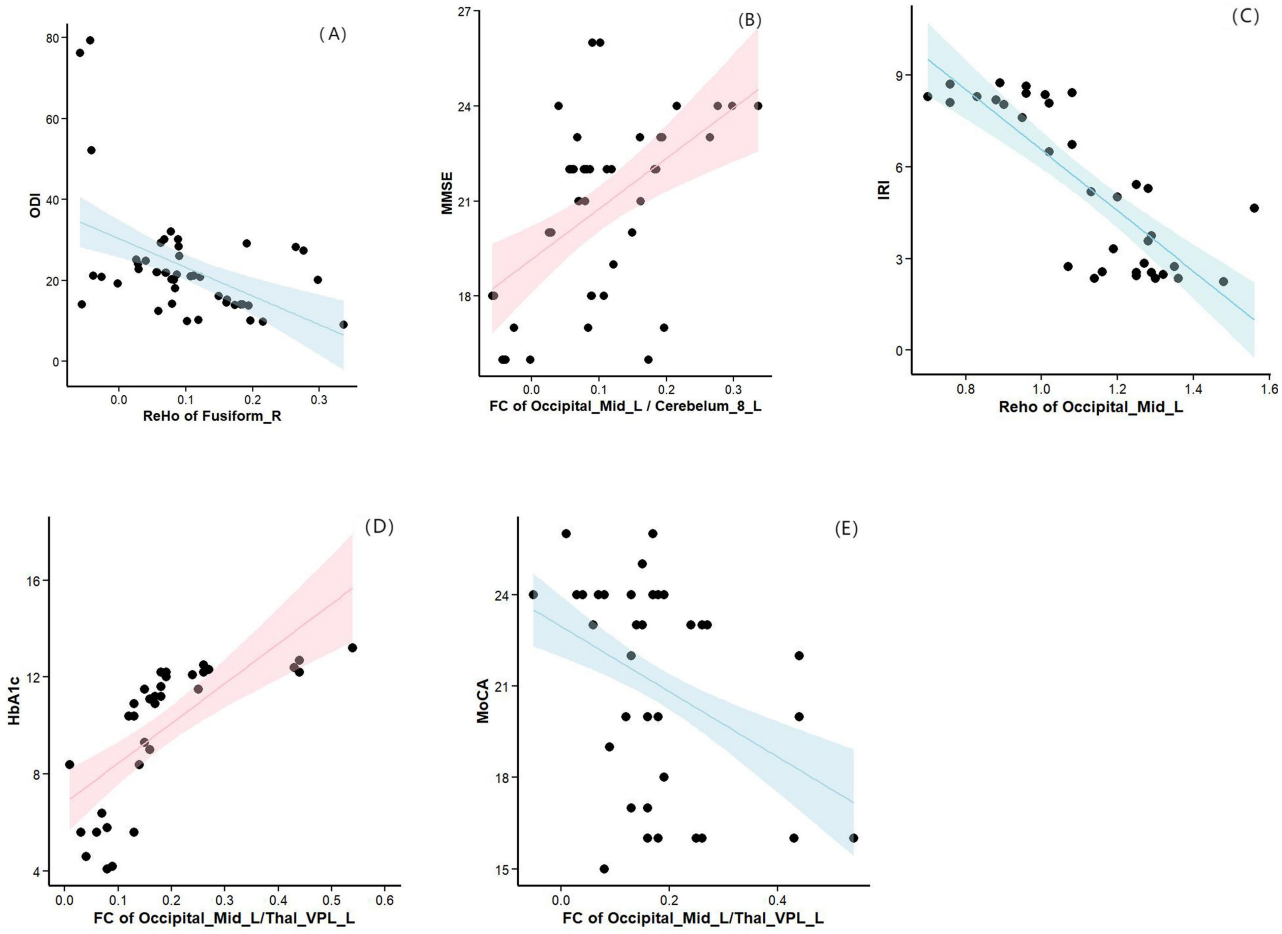
**(A)** Compared with the HCs group, the OSA group showed a significant negative correlation between ReHo in the right fusiform gyrus and ODI. **(B)** Compared with the HCs group, the OSA group showed a significant positive correlation between FC in the left cerebellum region 8 and MMSE scores. **(C)** Compared with the OSA group, patients in the OSA with T2DM group showed a significant negative correlation between ReHo in the left middle occipital gyrus and the IRI. **(D)** Compared with the OSA group, patients in the OSA with T2DM group showed a positive correlation between FC in the left thalamus and HbA1c. **(E)** Compared with the OSA group, patients in the OSA with T2DM group showed a negative correlation between FC in the left thalamus and MoCA scores. ReHo, Regional homogeneity; ODI, Oxygen Desaturation Index; MMSE, Mini-Mental State Examination; IRI, insulin resistance index; HbA1c, hemoglobinA1c; MoCA, Montreal Cognitive Assessment.

Partial correlation analysis was performed with sex, age, BMI, and years of education as covariates. Compared with the HCs group, the OSA group showed a significant negative correlation between ReHo in the right fusiform gyrus and ODI (*r* = −0.612, *p* = 0.000; Figure 5A), and a significant positive correlation between FC in the left cerebellum region 8 and MMSE scores (*r* = 0.392, *p* = 0.029; Figure 5B). Compared with the OSA group, patients in the OSA with T2DM group showed a significant negative correlation between ReHo in the left middle occipital gyrus and the IRI (*r* = −0.783, *p* = 0.000; Figure 5C), a positive correlation between FC in the left thalamus and HbA1c (*r* = 0.769, *p* = 0. 000; Figure 5D), and a negative correlation between FC in the left thalamus and MoCA scores (*r* = −0.478, *p* = 0.007; Figure 5E).

## 4 Discussion

This study employed a combined approach of ReHo and FC to investigate the impact of T2DM on brain function in OSA patients, and analyzed the correlation between brain region imaging differences, clinical variables, and cognitive scale scores. Our results indicated that, compared to the HCs group, the OSA group exhibited an increased ReHo in the left middle occipital gyrus, decreased ReHo in the right fusiform gyrus and left cerebellum 8 region, and reduced FC between the left middle occipital gyrus and left cerebellum 8 region, as well as between the right fusiform gyrus and left cerebellum 3 region. Furthermore, partial correlation analysis revealed that the ReHo of the right fusiform gyrus was significantly negatively correlated with ODI, while the FC between left middle occipital gyrus and the left cerebellum 8 region was significantly positively correlated with MMSE scores. Relative to the OSA group, the OSA with T2DM group showed a decreased ReHo in the left middle occipital gyrus and an increased FC between the left middle occipital gyrus and the left thalamus. Moreover, partial correlation analysis indicated that the ReHo of the left middle occipital gyrus was significantly negatively correlated with IRI, while the FC between the left middle occipital gyrus and the left thalamus was negatively correlated with MoCA scores and positively correlated with HbA1c levels. Overall, these findings reinforce the view of abnormal brain function changes in OSA patients and provide evidence that T2DM further affects brain function in OSA patients. This offers valuable insights into the neuro-pathological mechanisms of T2DM in OSA and supports the establishment of objective imaging biomarkers.

### 4.1 OSA–HCs

Compared with the HCs group, OSA patients exhibited decreased ReHo values in the right fusiform gyrus and the left cerebellum 8 region, while increased ReHo was observed in the left middle occipital gyrus. In addition, OSA patients showed significantly lower MoCA and MMSE cognitive scores than healthy controls, indicating marked cognitive impairment. This finding is consistent with previous studies, which have reported that OSA patients commonly experience persistent cognitive deficits, particularly in attention, episodic memory, and executive function (Bucks et al., 2017).

As an important component of the occipital visual processing area, the middle occipital gyrus is located in the higher visual cortex and is primarily involved in visuospatial processing and visual memory, serving as a key hub in the visual pathway (Villoslada and Alba-Arbalat, 2024). Previous studies have shown that the structural and functional integrity of the visual network has a significant impact on cognitive function. A study combining resting-state and task-based fMRI found that reduced functional connectivity within the visual network in patients with Alzheimer’s disease was closely associated with cognitive impairment, and disruption of this network may also promote the progression of mild cognitive impairment (MCI) (Huang et al., 2021). In addition, Yang et al. (2022) revealed from an electrophysiological perspective that OSA patients require significantly more time to process visual information, suggesting impaired visual perception, which may be a potential mechanism of OSA-related cognitive dysfunction. Supporting this view are brain network analysis results (Hayasaka et al., 2016) showing that the degree centrality (DC) of the left middle occipital gyrus is reduced in OSA patients, with significant negative correlations between DC and both the AHI and arousal index (AI). This suggests that functional connectivity impairment in the middle occipital gyrus is closely related to the severity of OSA and arousal frequency, with sleep fragmentation likely being a major cause of abnormal connectivity in this region. However, in the present study, increased ReHo was observed in this region among OSA patients, indicating enhanced local neural synchrony. We speculate that this change may reflect a neuroadaptive compensatory mechanism under chronic intermittent hypoxia, aimed at maintaining functional stability in this region.

The fusiform gyrus, located between the inferior temporal gyrus and the parahippocampal gyrus, is a key early visual cortex region, playing an important role in speech recognition, cognitive emotions, and emotional motor expression (Rhone et al., 2023; Picó-Pérez et al., 2017). A previous study (Kong et al., 2022) reported decreased functional connectivity between the ventral anterior insula and the fusiform gyrus in OSA patients. Additionally, the amplitude of low-frequency fluctuation (ALFF) in the right fusiform gyrus was found to be correlated with minimum SaO2, AI, and ODI, suggesting that this region is particularly sensitive to nocturnal hypoxia and sleep fragmentation. These findings imply that the fusiform gyrus may play a mediating role in OSA-related emotional disturbances and cognitive impairments. Our study indicates that OSA patients have reduced ReHo in the right fusiform gyrus, and the decreased ReHo is significantly negatively correlated with ODI, further supporting previous findings. OSA leads to changes in fusiform gyrus function due to nocturnal hypoxia and sleep fragmentation, which may contribute to emotional response deficits and cognitive impairments. This finding provides new evidence supporting the neuropathological basis of neuropsychiatric symptoms associated with OSA.

In recent years, the role of the cerebellum in OSA-related cognitive impairment has attracted increasing attention. Beyond its established function in motor control, the cerebellum is also extensively involved in sensorimotor integration, emotional regulation, learning, as well as higher-order cognitive processes such as attention and memory (Stoodley, 2011). Previous studies have demonstrated that the cerebellum is highly sensitive to hypoxia and ischemia, and its function can also be significantly affected by sleep deprivation (Kim et al., 2016; Li et al., 2024). Using resting-state fMRI, Park (2021) found that intermittent hypoxia may lead to impaired cerebellar network integration and functional connectivity, specifically showing reduced clustering coefficient and local efficiency of the left cerebellum within the salience network (SN), which was significantly associated with cognitive deficits. The SN serves as a critical “mediator” in the brain, primarily responsible for switching between the central executive network (CEN) and the default mode network (DMN). Damage to cerebellar function may weaken the SN’s regulatory role over these systems, thereby impairing task-switching abilities and contributing to the development and progression of mild cognitive impairment (MCI) (Deming et al., 2023). These findings provide a novel neural network perspective for understanding cognitive decline in OSA patients. In the present study, we further found that, compared with healthy controls, patients with OSA exhibited significantly reduced ReHo values in the left cerebellum 8 region. FC analysis also revealed decreased FC between the left middle occipital gyrus and the left cerebellum 8 region, as well as between the right fusiform gyrus and the left cerebellum 3 region. These results suggest abnormalities in both local neuronal activity and inter-regional connectivity in multiple cerebellar subregions associated with OSA. Functionally, cerebellum 8 region belongs to the neocerebellar cortex and is broadly involved in several intrinsic connectivity networks, including the CEN, DMN, SN, frontoparietal attention network, and language network. It supports cognitive functions such as working memory, episodic memory, spatial navigation, and attention (Habas, 2021). Although cerebellum 3 region has traditionally been regarded as mainly involved in motor coordination and balance, recent studies have shown that it also participates in cognitive regulation through cerebello-cortical loops, with consistent activation observed during working memory and attention tasks (Keren‐Happuch et al., 2012). Importantly, our partial correlation analysis further demonstrated a significant positive association between the FC of the left cerebellum 8 region and MMSE scores, reinforcing the notion that OSA may impair cognitive function by disrupting cerebellar-cortical connectivity. Taken together, cerebellar dysfunction may serve as a potential neuroimaging biomarker for early brain functional abnormalities and provide a promising target for early identification and clinical intervention in patients with OSA.

Unfortunately, compared to the HCs patients, we did not observe any brain function abnormalities in OSA with T2DM patients. One possible explanation is that certain brain regions in patients with T2DM may undergo metabolic adaptations. For example, they may maintain basic energy metabolism through insulin-independent glucose transport pathways, such as GLUT1 or GLUT3, which could account for the lack of significant differences in fMRI metrics (Kullmann et al., 2016). Additionally, the heterogeneity in the OSA group with T2DM (due to variations in OSA severity and the impact of T2DM treatment regimens) may have increased data variability, reducing statistical significance. Therefore, increasing the sample size in future studies may help detect corresponding brain function changes.

### 4.2 T2DM with OSA–OSA

Our findings indicate that, compared to the OSA group, the OSA with T2DM group showed further decreases in MoCA and MMSE scores, along with functional changes in certain brain regions, most notably a reduction in ReHo in the left occipital middle gyrus and an increase in FC between the left occipital middle gyrus and the left thalamus. The occipital middle gyrus, one of the most vulnerable areas in the brain to T2DM, plays a vital role in processing visual information related to visual cognition (Zhang et al., 2020). Its abnormal neural activity may also contribute to the pathophysiological mechanisms underlying cognitive impairments. Notably, we observed an increased ReHo value in the left middle occipital gyrus in the OSA-only group, which may represent an early compensatory mechanism of neural activity. However, in patients with comorbid T2DM, the ReHo value in this region was significantly decreased, suggesting that the compensatory mechanism in the middle occipital gyrus may be disrupted or exhausted under the combined influence of T2DM. Therefore, we speculate that T2DM may accelerate neuropathological changes in brain regions associated with OSA, leading to a decline in local neural synchronization. The exact pathophysiological mechanisms by which T2DM affects the central nervous system remain unclear, but most studies suggest that possible contributors include hyperglycemia, vascular complications, and insulin resistance (Lin et al., 2023). Insulin resistance, a characteristic pathophysiological defect in most T2DM patients, directly disrupts the insulin signaling pathway. The insulin signaling pathway plays a critical role in cognitive function by supporting various neuronal processes, including growth, survival, differentiation, migration, energy metabolism, gene expression, protein synthesis, cytoskeletal assembly, synapse formation, neurotransmitter function, and plasticity (Zheng et al., 2024). Therefore, damage to the insulin signaling pathway compromises the structural and functional integrity of the central nervous system, leading to the development of cognitive impairments. Our research revealed a negative correlation between the ReHo value in the left occipital middle gyrus and IRI, further confirming the critical role of insulin resistance in neurodamage among OSA patients. Further analysis in our study revealed a significant negative correlation between ReHo values in the left middle occipital gyrus and the IRI. This finding provides neuroimaging evidence for the association between insulin resistance and brain dysfunction, and further elucidates the exacerbating effect of T2DM on OSA-related brain abnormalities.

The thalamus, serving as a relay nucleus in the cerebral cortex, is anatomically and functionally divided into multiple distinct nuclei. These nuclei, through thalamocortical circuits, regulate information across various cortical regions, which explains the heterogeneity and complexity ofthalamic function (Wang et al., 2022). This complexity allows the thalamus to play a comprehensive role in cognition by regulating neuronal activity, supporting inter-region connectivity, promoting changes in network topology, enhancing neuronal variability, and modulating subtle and significant shifts in system-level arousal (Shine et al., 2023). Our research revealed that, in the OSA with T2DM group, the FC between the left occipital middle gyrus and the left thalamus was increased, and the FC value in the left thalamus showed a negative correlation with MoCA scores. This suggests that, compared to OSA patients, cognitive impairments were more pronounced in the OSA with T2DM group, with the body compensating through enhanced thalamic functional connectivity to sustain fundamental cognitive function. Several prior studies have also confirmed similar compensatory mechanisms. For instance, Wang found that selective thalamocortical hyperconnectivity helps maintain basic cognitive function in T2DM patients (Wang et al., 2022). Jing’s study indicated that enhanced FC between the bilateral thalamus and brain functional networks (including the visual network and DMN) in T2DM patients might serve as a compensatory mechanism for reduced brain region volumes associated with cognitive dysfunction (Jing et al., 2023). However, this hypothesis remains speculative due to the lack of supporting task-based fMRI or cognitive-behavioral data. Future studies may consider incorporating task paradigms involving executive control or visual processing, as well as analyses linking brain function with behavioral performance, to further verify its compensatory significance. Additionally, we observed a positive correlation between the FC value in the left thalamus and HbA1c levels. HbA1c reflects the percentage of glycated hemoglobin, which is an approximate indicator of the average plasma glucose concentration over the past 3 months and is regarded as the gold standard for evaluating diabetes treatment effectiveness. Research has shown that the abnormal accumulation of advanced glycation end-products (AGEs) due to hyperglycemia increases the production of reactive oxygen species (ROS), which in turn activates downstream APP-related pathways, enhances Aβ production, and upregulates NAD + -dependent deacetylase sirtuin 1 (Sirt1) and glucose-regulated protein 78 (GRP78), leading to neuronal cell death pathways and ultimately contributing to the onset of cognitive impairments (Kong et al., 2020). Additionally, GSK-3β plays a pivotal role in tau protein phosphorylation and in insulin signaling. Disruptions in insulin signaling that regulate the GSK-3β pathway in diabetes, combined with hyperglycemia, may trigger tau phosphorylation and subsequent cleavage, which increases the risk of cognitive disorders, such as Alzheimer’s disease, in diabetic patients (Wee et al., 2023). Finally, hyperglycemia can trigger apoptotic processes, including caspase activation, which leads to tau protein cleavage and increases neuronal susceptibility to Aβ-induced damage (Kim et al., 2013). These findings underscore the critical role of hyperglycemia in mediating cognitive impairment in OSA patients.

It is worth noting that although the comorbidity of T2DM and OSA may exacerbate brain functional impairments, this process may be, to some extent, reversible. Previous research has shown that good glycemic control—such as maintaining HbA1c within an optimal range—is closely associated with preserved cognitive function and improved cerebral metabolism (Sanchez-Rangel et al., 2022; Zhao et al., 2025). Therefore, future studies are warranted to investigate whether glycemic control interventions could help ameliorate functional connectivity abnormalities in patients with T2DM and OSA, thereby providing evidence to support more targeted clinical management strategies.

### 4.3 Limitation

This study has several limitations. First, it only included moderate to severe OSA patients, and the potential impact of mild OSA on diabetes incidence cannot be ruled out. Future cohort studies with more robust designs are needed to explore this finding, as mild OSA is more prevalent than moderate to severe OSA and has greater public health significance. Secondly, we observed functional abnormalities in the occipital gyrus in both the OSA and OSA with T2DM groups. Since the occipital gyrus plays a crucial role in visual processing, future research should incorporate visual tests to assess patients’ ability to process visual information. Furthermore, potential bias may exist in the use of hypoglycemic medications among T2DM patients in this study. Variations in drug type and dosage may influence brain functional activity. Future studies should collect more detailed medication information and control for both drug categories and dosages to minimize such confounding effects. Lastly, although the sample size in this study was determined with reference to previous literature and practical feasibility, the number of participants in the OSA with T2DM group was relatively small, which may have resulted in insufficient statistical power to detect subtle brain functional alterations. Future research should aim to expand the sample size and incorporate multicenter data to enhance the robustness of the findings.

## 5 Conclusion

This study employed rs-fMRI ReHo combined with FC analysis to investigate the impact of T2DM on brain function in OSA patients, as well as the correlations between brain function abnormalities, IRI, HbA1c, and neuropsychological test scores. This study revealed the disruption of neural function in OSA patients with T2DM, providing additional evidence to elucidate the neural mechanisms through which T2DM affects brain function in OSA patients.

## Data Availability

The raw data supporting the conclusions of this article will be made available by the authors, without undue reservation.

## Footnotes

^1^ http://www.restfmri.net
